# Mapping Neurophysiological Patterns in Carpal Tunnel Syndrome: Correlations With Tinel’s and Phalen’s Signs

**DOI:** 10.7759/cureus.58168

**Published:** 2024-04-13

**Authors:** Sanjeev K Saggar, Richa G Thaman, Gurinder Mohan, Dinesh Kumar

**Affiliations:** 1 Physiology, Sri Guru Ram Das Institute of Medical Sciences and Research, Amritsar, IND; 2 Physiology, Medical Education, Sri Guru Ram Das Institute of Medical Sciences and Research, Amritsar, IND; 3 General Medicine, Sri Guru Ram Das Institute of Medical Sciences and Research, Amritsar, IND; 4 Neurology, Sri Guru Ram Das Institute of Medical Sciences and Research, Amritsar, IND

**Keywords:** compression-induced neuropathy (cin), tinel’s and phalen’s sign, median nerve, nerve conduction study (ncs), carpel tunnel syndrome (cts)

## Abstract

Background

This study aimed to observe the neurophysiological severity grading of carpel tunnel syndrome (CTS) using nerve conduction studies (NCSs) and the correlation between Tinel’s and Phalen’s signs.

Methodology

In this cross-sectional study, 240 patients of CTS were enrolled. NCSs were conducted in 480 hands. Various variables such as distal latency, amplitude, and nerve conduction velocity in both sensory and motor median nerves were recorded. The provocative tests capable of reproducing patients’ symptoms such as Phalen’s test and Tinel’s test were performed on all 480 hands studied.

Results

Neurophysiological variables were affected in 449 out of 480 hands. Tinel’s sign was observed in 59% of cases (265 hands) while Phalen’s sign was positive in 37.2% (167 hands) of cases. Severity grading of CTS based on neurophysiological variables resulted in Grade I (mild) in 202 hands, Grade II (mild to moderate) in 56 hands, Grade III (moderate) in 39 hands, and Grade IV (severe) in 152 hands. Provocative tests (Tinel’s and Phalen’s) used for the diagnosis of CTS were positive in 68 hands (36.66%) and 26 hands (12.8%), respectively, in mild Grade I. However, as the CTS severity grade increased, the provocative test success rate also increased simultaneously. In severe Grade IV CTS, Tinel’s and Phalen’s tests were positive in 134 (88.1%) hands and 94 (61.8%) hands, respectively.

Conclusions

This study underscores the unreliability of Tinel’s and Phalen’s signs as screening methods for CTS severity. With moderate sensitivity and specificity, NCSs are deemed essential for confirming CTS diagnosis and assessing severity, especially in mild cases that might be mistakenly perceived as normal hands by consultants.

## Introduction

Carpal tunnel syndrome (CTS) is a compression neuropathy caused by a combination of factors that increase pressure on the median nerve and tendons in the carpal tunnel. It may cause numbness, pain, and tingling along the lateral aspect of the palmer surface of the hand. It usually affects the lateral three-and-a-half fingers. CTS was reported first by W R Brain in 1947 in six patients engaged in repetitive work [1].

It is more common in women than men (139 per 100,000 persons per year for men and 506 per 100,000 persons per year for women) [2,3]. It usually involves the dominant hand first [4] and can manifest at any age; however, the peak incidence is around 40 to 60 years [5]. It usually accounts for a higher number of absentee days away from work than other work-related musculoskeletal disorders [6]. Patients may exhibit frequent night awakenings due to pain and paresthesia in the hand, and it is relieved by shaking hands. CTS exhibits higher prevalence in certain professions that involve repetitive hand movements, especially wrist flexion, repetitive grasping, or pinching of objects. CTS is clinically diagnosed and is confirmed by abnormal electrophysiological tests [7].

It is crucial to diagnose CTS at an early stage to exclude other causes and further prevent median nerve damage [7,8]. It has been observed that the prognosis becomes worse if clinical manifestations of CTS are severe. A variety of median nerve motor and sensory tests are available to establish median neuropathy in patients of CTS [9]. The relationship between electrophysiological variables and clinical grading of CTS has been demonstrated in a few previous studies [10].

This study aimed to study the neurophysiological pattern and severity grading of CTS in patients visiting a tertiary care medical institute in Amritsar, India. The study aimed to learn the demographic and electrophysiological profile of patients and tried to rationalize the relationship between electrophysiological diagnosis or variables and clinical grading of CTS. The study was conducted to observe and analyze the correlation between provocative tests, namely, Tinel’s sign and Phalen’s sign, the clinical primary tests which are subjective (based on patient response), and neurophysiological variables determined by nerve conduction study (NCS).

## Materials and methods

This prospective, cross-sectional study was conducted in the Department of Physiology and Nerve Conduction Lab (under the Department of Medicine (Neurology)) in a tertiary care medical institute in Amritsar, Punjab. A total of 240 patients (480 hands) who were clinically confirmed with CTS were included in this study. The clinical diagnosis of CTS was based on the following criteria given by Vogt et al. [11]: (a) activity-related pain or nocturnal pain or dysesthesia in hand; (b) isolated atrophied abductor pollicis brevis muscle; (c) positive Tinel’s or Phalen’s signs; (d) sensory impairment or deficit or reduced two-point discrimination in median nerve distribution. Patients were suspected of having CTS if they complained of painful dysesthesia in the sensory area of the median nerve along with one criterion from b to d.

The clinical features and neuro-laboratory features were recorded in a prescribed proforma. This prospective study was conducted from July 2018 to July 2022 among cases of CTS reported in the Outpatient Department of Medicine, Neurology, and Orthopedics. Each wrist was considered separately for clinical and neurophysiological diagnosis. A total of 480 hands were studied. A nerve conduction velocity (NCV) machine (MS Aleron) from Recorder and Medicath System (P) Ltd. was used to perform NCS. The following nerve conduction tests were performed in all patients: (1) median motor and sensory distal latency; (2) median motor and sensory amplitude; and (3) median motor and sensory conduction velocity.

In this study, we used disc recording electrodes measuring 1 cm for mixed nerve studies, and sensory studies were conducted using ring electrodes. Percutaneous supramaximal response/stimulus was used for NCSs using the same instrument. The pulse duration for the mixed and sensory nerves was kept at 0.05/0.1 ms, while a pulse duration of 0.2/0.5 ms was used for stimulating the motor nerve. The filters were set at 20 Hz and 2 KHz. A ground electrode was placed between the stimulating and recording electrodes. Patients were graded according to severity into mild, mild to moderate, moderate, and severe according to the following criteria given by Hermann and Logigian [12]: (1) mild: prolongation of median distal motor and sensory latency alone; (2) mild to moderate: latency prolongation with mild reduction of sensory nerve action potential (SNAP); (3) moderate: latency prolongation along with a moderate reduction in SNAP or compound muscle action potential (CMAP); (4) severe: unrecordable median SNAP or severe reduction of CMAP.

In this study, 240 patients were independently administered Phalen’s and Tinel’s tests. In Phalen’s test, research participants were asked to sit and rest their elbows on the table while holding both forearms in vertical alignment with the volar surface aligned medially. Participants were then instructed to let their wrists relax into full palmar flexion, and a positive response was defined as the reproduction of symptoms in the median distribution of the palmar hand within 60 seconds [13].

The Tinel’s sign was performed on participants by tapping the wrist crease over the median nerve with a tendon hammer. A positive response was defined as a sensation of tingling in the distribution of the median nerve in the hand. The evocative test was judged as positive when each test gave a positive result [14].

Inclusion and exclusion criteria

We included patients aged 15-75 years who were clinically diagnosed or suspected of having CTS. Patients with underlying peripheral polyneuropathy were excluded from the study. CTS patients who did not provide informed consent to participate in the study were excluded. Moreover, patients with electronically or mechanically activated implants, i.e., cardiac pacemakers, were also excluded.

The study was approved by the Institutional Ethics Committee as per the norms and was conducted in compliance with the Declaration of Helsinki. Study participants received information about the study and instructions regarding additional tests they would undergo. Written informed consent was obtained before enrolling participants in the study.

Statistical analysis

Statistical analysis for the present study was done using SPSS version 26 (IBM Corp., Armonk, NY, USA). The assessment of variable distribution played a critical role in choosing a suitable statistical method. Consequently, the Shapiro-Wilk test indicated a significant departure from normality in the data distribution (p < 0.01). In response to these findings, a non-parametric test was applied, and the data were summarized using the median and interquartile range. To find the difference between the two groups, the Mann-Whitney U test was applied. For statistical evaluations, p-values <0.05 were considered significant, and p-values <0.001 were considered highly significant. Sensitivity, determined by the ratio of positive Tinel’s/Phalen’s test results among hands with confirmed CTS through NCS, and specificity, evaluated by the ratio of negative Tinel’s/Phalen’s test results among hands without electrodiagnostically confirmed CTS, were used to compute positive and negative predictive values.

## Results

A total of 240 patients with CTS were studied, of whom 181 were females while 59 were males. The female-to-male ratio was 3.06:1. Patients had a mean age of 49.18 ± 11.92 years (Table 1). Of the 480 hands analyzed, 449 were found to be symptomatic.

**Table 1:** Distribution of patients and hands affected based on gender.

Gender	Number of patients	Percentage	Number of hands	Percentage
Female	181	75.4	344	77
Male	59	24.6	105	23
Total	240	100.0	449	100

In our study, the 41-50-year age group had 67 (27.9%) patients while the 51-60-year age group had 70 (2 9.2%) patients. In our study, the 40-60-year age group was most vulnerable to CTS (Table 2).

**Table 2:** Distribution of patients based on age group.

Age group (years)	Number of patients	Percentage
≤30	16	6.7
31–40	46	19.2
41–50	67	27.9
51–60	70	29.2
>60	41	17.1
Total	240	100.0
Age (mean ± SD)	49.18 ± 11.92	

Bilateral symptoms of CTS syndrome were observed in 209 (87.1%) patients. The mean duration of symptoms was 7.55 ± 5.32 months (Table 3).

**Table 3:** Distribution of patients based on the duration of disease.

Duration (months)	Number	Percentage
>6	88	36.7
6–12	134	55.8
>12	18	7.5
Total	240	100.0
Duration of disease (mean ± SD)	7.55 ± 5.32	

**Figure 1:**
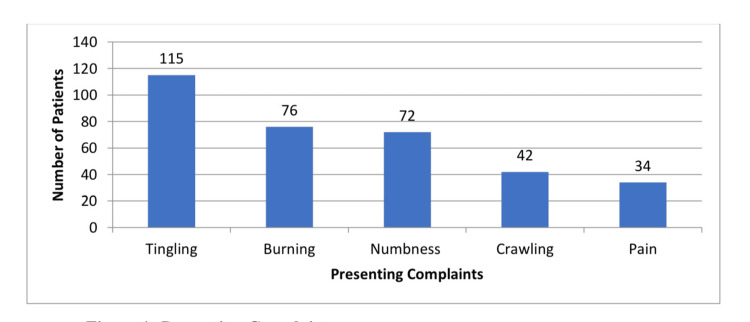
Presenting complaints in carpel tunnel syndrome patients.

The main complaint of 240 patients was tingling (n = 115, 47.9%), followed by burning (n = 76, 31.7%) numbness (n = 72, 30%)c, crawling/formication (n = 42, 17.5%), and, lastly, pain sensation (n = 34, 14.2%) (Figure 1).

Of the 480 hands, 449 were affected or symptomatic. Of the 449 symptomatic hands, Grade I (mild) was seen in 202 (45%) hands, Grade II (mild to moderate) in 56 (12.5%) hands, Grade III (moderate) in 39 (8.7%) hands, and Grade IV (severe) in 152 (33.9%) hands. Demyelinating median nerve injury was observed in 45% of hands, severe axonal median nerve injury was observed in 33.9%, and demyelinating and axonal injury/neuropathy was observed in 21.2% of hands.

**Figure 2:**
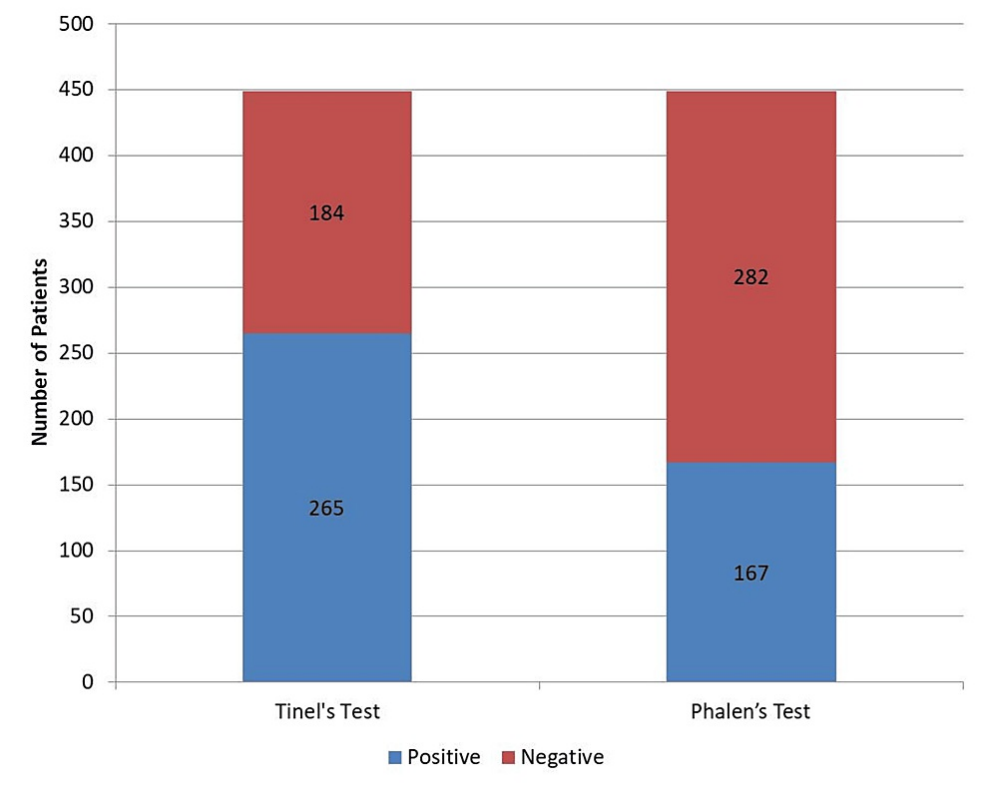
Presence of Tinel’s test and Phalen’s test in the study participants.

Tinel’s sign and Phalen’s maneuver are classically associated with CTS. In this study, of the 449 symptomatic CTS hands, Tinel’s sign was positive in 265 (59%) hands whereas Phalen’s sign was positive in 167 (37.2%) hands. A comparison of NCSs of hands with positive Tinel’s sign and symptomatic hands without Tinel’s sign is shown in Figure 2.

In hands with a positive Tinel’s sign, the motor amplitude (CMAP) was 11.6 (7-14.78) mV and sensory amplitude was 26.38 (16.8-35.7) µV, while in symptomatic hands with a negative Tinel’s sign, the motor amplitude was 14.25 (11.2-17.43) mV and sensory amplitude was 34.75 (20.4-44.2) µV. Sensory latency in hands with a positive Tinel’s test was 3.64 (2.88-3.88) ms, whereas sensory latency in hands with a negative Tinel’s test was 2.63 (2.33-3.62) ms. Motor latency in hands with a positive Tinel’s sign was 4.01 (3.45-4.77) ms, whereas in hands with a negative Tinel’s sign, motor latency was 3.54 (3.02-3.85) ms. The observations of NCS between negative and positive Tinel’s test were found to be highly significant (p < 0.001), as per the Mann-Whitney U test (Table 4).

**Table 4:** Neurophysiological/Nerve conduction study in hands with Tinel’s sign present and absent.

	Tinel’s test present		Tinel’s test absent		P-value
	Number	Median (IQR)	Number	Median (IQR)	
Motor latency (ms)	264	4.01 (3.45–4.77)	184	3.54 (3.02–3.85)	<0.001
Motor amplitude (mv)	264	11.6 (7–14.78)	184	14.25 (11.2–17.43)	<0.001
Motor nerve conduction velocity (m/s)	264	48.87 (43.34–53.74)	184	52.68 (48.87–56)	<0.001
Sensory latency (ms)	131	3.64 (2.88–3.88)	166	2.63 (2.33–3.62)	<0.001
Sensory amplitude (µv)	131	26.38 (16.8–35.7)	166	34.75 (20.4–44.2)	<0.001
Sensory nerve conduction velocity (m/s)	131	34.27 (29.02–42.61)	166	44.14 (37.54–49.77)	<0.001

In hands with a positive Phalen’s test, the motor amplitude (CMAP) was 11.3 (7-14.5) mV, motor distal latency was 4.4 (3.54-4.85) ms, sensory distal latency was 3.71 (3.23-3.92) ms, and sensory amplitude (SNAP) was 19.4 (14.25-32.8) µV. In symptomatic hands with a negative Phalen’s test, motor amplitude (CMAP) was 13.7 (10.8-16.55) mV, motor distal latency was 3.6 (3.12-4.12) ms, sensory latency was 2.75 (2.38-3.67) ms, and sensory amplitude was 33.4 (19.68-44.2) µV. We found a statistically highly significant (p < 0.001) difference in the observations of NCS between negative and positive Phalen’s sign, as per the Mann-Whitney U test (Table 5).

**Table 5:** Neurophysiological variables/nerve conduction study in hands with Phalen’s test present and absent.

	Phalen’s test present		Phalen’s test absent		P-value
	Number	Median (IQR)	Number	Median (IQR)	
Motor latency (ms)	167	4.4 (3.54–4.85)	281	3.6 (3.12–4.12)	<0.001
Motor amplitude (mv)	167	11.3 (7–14.5)	281	13.7 (10.8–16.55)	<0.001
Motor nerve conduction velocity (m/s)	167	48.87 (43.33–53.74)	281	51.66 (47.14–55.44)	<0.001
Sensory latency (ms)	73	3.71 (3.23–3.92)	224	2.75 (2.38–3.67)	<0.001
Sensory amplitude (µv)	73	19.4 (14.25–32.8)	224	33.4 (19.68–44.2)	<0.001
Sensory nerve conduction velocity (m/s)	73	33.03 (28.13–40)	224	42.63 (34.27–47.85)	<0.001

**Figure 3:**
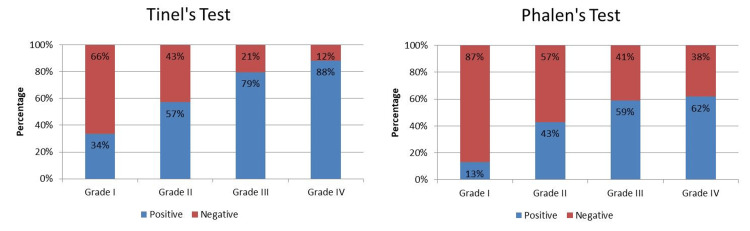
Distribution of hands based on grades along with the presence of Tinel’s and Phalen’s signs.

In Grade I CTS symptomatic hands (n = 202), Tinel’s sign was positive only in 68 (34%) cases. In Grade II CTS (n = 56), Tinel’s sign was positive in 32 (57%) cases, whereas, in Grade III (n = 39), Tinel’s sign was positive only in 31 (79.4%). In Grade IV (n = 152), only 134 (88.1%) cases showed a positive Tinel’s positive. Therefore, NCSs or neurophysiological variables are affected in mild CTS and can be detected and treated while clinical experiments and history may sometimes miss the diagnosis of early onset of CTS (Figure 3).

Similarly, Phalen’s sign was positive in 26 (12.8%) cases in Grade I (n = 202) of the 202 CTS symptomatic hands. In Grade II, 24 (42.8%) hands showed a positive Phalen’s test out of 56 symptomatic CTS hands. In Grade III, 23 (58.9%) hands showed a positive Phalen’s test out of 39 CTS symptomatic hands. In Grade IV, 94 (61.8%) hands showed a positive Phalen’s test out of 152 symptomatic hands (Figure 3).

**Figure 4:**
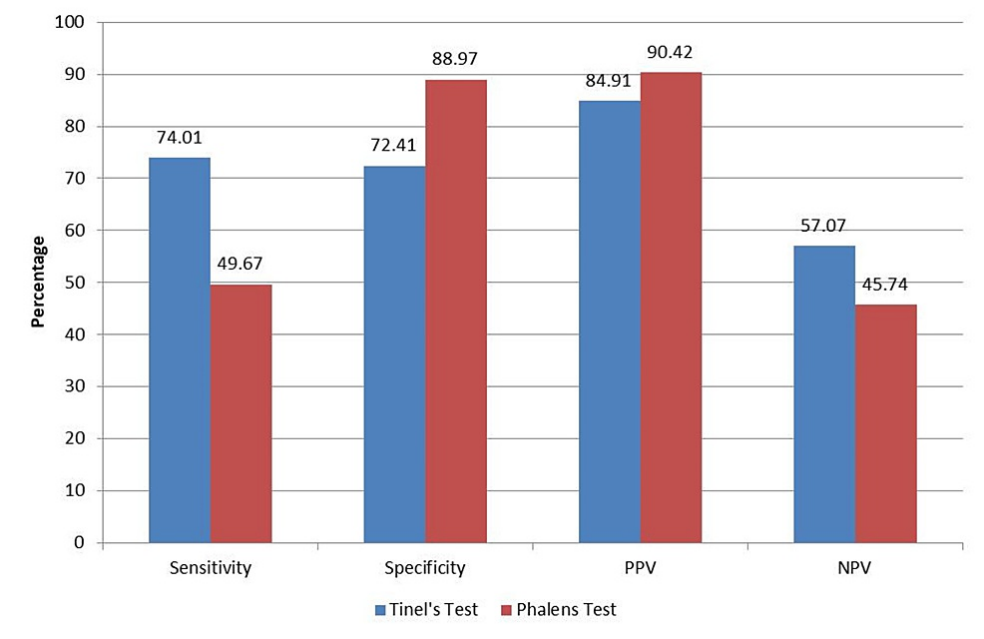
Sensitivity, specificity, positive predictive value (PPV), and negative predictive value (NPV) of Tinel’s and Phalen’s tests for carpel tunnel syndrome.

We further calculated the sensitivity and specificity of Tinel’s and Phalen’s signs. Tinel’s test demonstrated a sensitivity of 74.01% and specificity of 72.41%. Phalen’s test exhibited a sensitivity of 49.67% and a specificity of 88.97%. The positive predictive value (PPV) of Tinel’s and Phalen’s tests was 84.91% and 90.42%, respectively. The negative predictive value (NPV) of Tinel’s and Phalen’s tests was 57.07% and 45.74%, respectively (Figure 4).

## Discussion

There is no gold standard diagnostic test for CTS. Clinical history and clinical assessment alone based on signs and symptoms, along with experimental conduction of Tinel’s sign and Phalen’s maneuver, is not completely successful in accurately diagnosing CTS. The objective diagnosis of CTS is commonly established by various electrodiagnostic tests. However, in the absence of electrodiagnostic studies, symptom characteristics along with physical examination are likely to result in greater misclassification of disease status [15].

The median distal motor latency, median sensory latency test, and CMAP (median motor amplitude and median sensory nerve amplitude) are the basic electrodiagnostic parameters used in the diagnosis of CTS. In this study, female patients predominated in our study, and out of 181 female patients, 120 were housewives engaged in repetitive household activities (i.e., flour kneading). Useful information is derived from electrophysiological studies regarding the quantitative assessment of the neurophysiological severity of CTS[16]. Generally, the median distal latency (motor) and the median distal latency (sensory) are accepted as the standard and conventional electrophysiological parameters for diagnosing and assessing the function of the median nerve in CTS. NCSs in CTS cases are used to assess both sensory and motor median nerve fibers [17,18]. Electrophysiological severity classification is a useful tool to evaluate parameters on simple, analytic, and single scales. Moreover, the age of patients and clinical findings were found to be related to neurophysiological abnormalities. Furthermore, the majority of advanced cases occurred in older patients [19,20]. In our study, the mature age group of patients, i.e., above 40 years of age, was observed to be the most vulnerable to CTS.

Primary provocative tests are subjective tests based on patients’ responses. This leaves room for error in the assessment of the severity of CTS. The use of NCS and electromyography is more evidence-based and does not depend on patients’ clinical responses [21,22]. In this study, an effort was made to study neurophysiological variables determined by electrodiagnostic tests, i.e., SNAP, CMAP, distal latency (sensory), and distal latency (motor), which were compared in hands with positive Tinel’s sign and negative Tinel’s sign (clinical assessment). It was observed that neurophysiological variables, i.e., CMAP and SNAP, were statistically lower in hands with positive Tinel’s signs compared to negative Tinel’s signs. This establishes that neurophysiological variables are affected first before clinical signs and symptoms manifest in patients; therefore, a combined approach using clinical and neurophysiological approaches must be adopted for establishing median nerve entrapment at the wrist.

One important observation from the present study is that in Grade I patients, Tinel’s sign was absent in 66.3% of the hands and Phalen’s sign was absent in 87.1% of the hands. Therefore, a clinical consultant has a high chance of misdiagnosing mild cases of CTS without NCS. As the severity of CTS increased, the provocative test positivity increased. In Grade IV, Tinel’s sign was absent in 11.8% of the hands while Phalen’s sign was absent in 38.1% of the hands. The SNAP becomes unrecordable as CTS becomes more severe, while the distal motor latency is unaffected in early CTS and may become unrecordable at severe extremes.

In comparison to our study, Bland proposed that NCS does not define the presence of CTS. We need to analyze the results with caution. It may require an in-depth understanding of the relationship between NCS findings, examination results, and treatment outcomes. Using the interpretation of one test will decrease the correctness of the diagnosis of CTS and multiple criteria need to be used [23].

This study indicated that provocative tests aimed at reproducing patients’ symptoms such as Phalen’s test and Tinel’s test cannot alone serve as independent diagnostic tools for CTS. These provocative tests are quite popular in clinical settings; however, this study emphasizes the importance of NCS in grading the severity of CTS, as well as in diagnosing CTS, especially in early and moderate severity of CTS, where a substantial number of Tinel’s and Phalen’s tests were negative. Neurophysiological variables, i.e., CMAP, SNAP, NCV (sensory), NCV (motor), distal latency (sensory), and distal latency (motor), were statistically highly significant in positive Tinel’s test compared to negative Tinel’s test in CTS hands. Similar statistically significant results were observed for neurophysiological variables when Phalen’s test-positive CTS hands were compared to Phalen’s test-negative CTS hands. This study supports that NCS is an imperative tool for the diagnosis of CTS, especially in mild and mild to moderate CTS cases, i.e., just at the onset or beginning of CTS or beginning of the distortion in neurophysiological variables which is difficult to assess by clinical tools.

Another study reiterates that including provocative test results in the NCS findings improves the overall diagnosis of CTS. These tests identified patients with severe CTS. Sensorimotor impairment due to CTS affects the working of the hands [24]. In our study, the positivity of provocative tests increased with the severity of the CTS grade, as found by NCS. Another study by Izadi et al. did not find a correlation between provocative tests and NCS findings. As seen in our study, this study also found that sensory and motor parameters were significantly correlated with clinical grades. It was also concluded that provocative tests are not reliable alone for the diagnosis of CTS [25].

Tinel’s test showed a sensitivity of 74.01% in effectively identifying CTS with a specificity of 72.41% to minimize false positives. Phalen’s test had a 49.67% sensitivity in detecting CTS with a high specificity of 88.97%, reducing false positives. The PPVs for Tinel’s and Phalen’s tests were 84.91% and 90.42%, respectively, signifying reliability in confirming CTS. The NPVs for Tinel’s and Phalen’s tests were 57.07% and 45.74%, respectively, suggesting moderate accuracy in ruling out CTS. Zhang et al. [26] reported a sensitivity and specificity of 47% and 56% for Tinel’s test and 50% and 33% for Phalen’s test, respectively. A meta-analysis by Ozdag et al. [27] revealed median sensitivity and specificity for Tinel’s sign as 59% and 80% and for Phalen’s sign as 70% and 80%, respectively. These findings highlight considerable variability in sensitivity and specificity among tests for CTS in various studies and prior literature. The tests may be helpful when interpreted in the appropriate clinical context. However, the sensitivity and specificity of these tests are modest at best and should be interpreted with caution in the absence of electrodiagnostic studies.

## Conclusions

Individuals suffering from CTS experience sensorimotor impairments that disrupt hand dexterity. This study asserts that conventional clinical signs and associated provocative tests, such as Tinel’s sign and Phalen’s sign, fall short of accurately assessing the severity and diagnosis of CTS. Instead, the study advocates for the indispensability of NCSs to confirm the diagnosis and determine the severity of CTS. Provocative tests are deemed more effective in detecting CTS in moderate-to-severe cases. Consequently, abnormal neurophysiological variables identified through NCS play a crucial role in grading mild CTS cases, which may be mistakenly perceived as normal hands in a clinical setting. The discriminatory capability of provocative tests for different levels of CTS severity is limited compared to NCS. The diagnostic accuracy of these tests is particularly constrained in mild and mild-to-moderate cases, necessitating the use of NCS for precise diagnoses.

The study proposes a holistic diagnostic model integrating NCS with provocative tests, clinical signs, and symptom history for early, mild-to-moderate, and severe CTS cases. Specifically, it recommends conducting NCS for early CTS patients and those undergoing surgery for severe CTS to validate the diagnosis. Utilizing the results rationally requires a profound understanding of the interplay among clinical findings, NCS, and provocative test results to arrive at a highly accurate diagnosis.
